# Virginia Memory Project: Using the Healthy Brain Initiative Roadmap to design a statewide dementia registry

**DOI:** 10.1002/alz.14478

**Published:** 2025-01-16

**Authors:** Annie Rhodes, Ashley Staton, Evan French, Andrea Price, Brian Battle, Catherine MacDonald, Kimberly Ivey, Faika Zanjani, Rachel Coney, Melicent Miller, Meghan Farkas, Lana Sargent, Daniel Bluestein

**Affiliations:** ^1^ Virginia Center on Aging College of Health Professions Virginia Commonwealth University Richmond Virginia USA; ^2^ Wright Center for Clinical and Translational Research Virginia Commonwealth University Richmond Virginia USA; ^3^ Healthy Brain Virginia Program Division of Prevention and Health Promotion Virginia Department of Health Richmond Virginia USA; ^4^ Office of Practice and Community Engagement School of Nursing Virginia Commonwealth University Richmond Virginia USA

**Keywords:** CDC BOLD ACT: Virginia, dementia innovation, dementia policy, dementia registry, epidemiology, health equity, Healthy Brain Initiative

## Abstract

**INTRODUCTION:**

The Virginia Memory Project (VMP) is a statewide epidemiological registry for Alzheimer's disease and related disorders (ADRD) and other neurodegenerative conditions. It aims to support dementia research, policy, and care by leveraging the Centers for Disease Control (CDC) Healthy Brain Initiative (HBI) Roadmap.

**METHODS:**

To capture comprehensive data, the VMP integrates self‐enrollment and automatic enrollment using Virginia's All‐Payer Claims Database (APCD). It also adapts Behavioral Risk Factors Surveillance Survey (BRFSS) modules for self‐reported cognitive and caregiving data, offering connections to research, clinical services, and education.

**RESULTS:**

Virginia successfully codified the VMP in the 2024 general assembly session.

**DISCUSSION:**

The VMP demonstrates a novel approach to Alzheimer's Disease and Related Disorders (ADRD) surveillance by combining traditional registry functions with community engagement and workforce development. Future efforts will focus on increasing enrollment, especially among underrepresented groups, to enhance data‐driven dementia policy and care in Virginia.

**Highlights:**

- Integrated the Healthy Brain Initiative (HBI) domains into the newest statewide epidemiological dementia registry in the Commonwealth of Virginia.
- Collected data and identified gaps in the current research related to dementia and Alzheimer's related diseases.
- Aimed to mitigate barriers to dementia registry enrollment by identifying significant underdiagnosis and underrepresentation of racial and ethnic minority groups.
- Developed solutions to alleviate the current data and enrollment disparities and to connect individuals to research, physicians, and community groups.

## 1 INTRODUCTION

In March 2024, The Virginia Memory Project (VMP) was codified by the General Assembly and the Governor of Virginia, establishing the newest statewide dementia registry in the United States. The VMP catalogs diagnoses of Alzheimer's disease and related disorders (ADRD), caregiving, subjective cognitive impairment (SCI), loneliness, social isolation, and other neurodegenerative conditions (such as Parkinson's disease).

With a unique structure allowing self and auto‐enrollment, the VMP is a resource hub, providing connections to research trials, clinical and social services, and education. The VMP leverages the Centers for Disease Control (CDC) Healthy Brain Initiative (HBI) Roadmap to design, launch, and maintain an epidemiological registry and influence health policy.

The HBI Roadmap has four domains: (1) measure, evaluate, and utilize data; (2) build a diverse and skilled workforce; (3) engage and educate the public; and (4) strengthen partnerships and policies.^1^ The VMP fulfills these domains by integrating multiple data sources to analyze trends, estimate prevalence, champion advocacy initiatives, and provide resources.

The VMP is housed within Virginia Commonwealth University's Virginia Center on Aging and was publicly launched in 2022 via a sub‐award from the Virginia Department of Health (VDH) and CDC: Building our Largest Dementia Infrastructure Act (BOLD).^2^ In the 2024 state legislative session, the VMP was codified by the General Assembly and signed by the Governor.^3^ Codification is a monumental step in supporting a coordinated dementia response in Virginia. It ensures consistency in ADRD data availability, equipping policymakers with information on various topics including risk factors and social determinants of health. It also supports overall data‐driven policy creation and resource allocation for an ADRD response.

This short report aims to (1) provide readers with information on the advantages and limitations of dementia registries while educating readers on the VMP and (2) delineate a model for integrating the HBI framework into statewide ADRD registry operations to support coordinated dementia programming.

**RESEARCH IN CONTEXT**

1. **Systematic review**: The authors searched and reviewed the literature using traditional sources (e.g., PubMed, National Institutes of Health [NIH] Journals Library) to identify abstracts with content related to “Alzheimer's Disease and Related Disorders” AND “disease registries”. Although these topics appear in the literature body, significant challenges arise related to progress in the treatment and prevention of dementia, specifically due to difficulties in registry enrollment and the identification of dementia diagnoses in underserved populations.
2. **Interpretation**: Although not widely used across the United States, epidemiological dementia registries are imperative to identifying dementia risk factors and assisting in the development of community‐focused policy, resource allocation, and program evaluation. Our findings led to the proposed solutions, which are consistent with the Healthy Brain Initiative (HBI) Roadmap to maintain a data‐driven and community‐focused dementia registry through the Virginia Memory Project (VMP).
3. **Future directions**: This article proposes a framework and solutions to bridging the gap between those who are experiencing dementia, as well as their caregivers, and the data being collected to represent these individuals. The VMP strives to mediate these barriers to enrollment and data collection by combining information sources, engaging in education and advocacy, and being a responsive resource. In addition, multidisciplinary collaboration is essential to help identify those who need assistance and allow for access to data to enhance the traditional approaches to dementia research and prevention.

## 2 BACKGROUND AND NATURE OF THE PROBLEM

### 2.1 Silos in dementia registries

Dementia registries are utilized globally.^4^ Simply defined, a registry compiles data focused on the outcomes of a defined patient population.^5^ Epidemiological disease registries are public health tools for tracking disease prevalence and outcomes, providing consistent population‐level data. Registries estimate the prevalence of chronic and infectious diseases and project subsequent health care demand.^5^, ^6^ Given the global prevalence of dementia, which is projected to increase to 152.8 million by 2050,^7^ and the importance of prevention efforts, in the United States, dementia registries are an emerging but underutilized strategy for informing policy and resource response.^4^, ^6^, ^8^

Dementia registries typically focus on one of three aims: (1) enrolling participants in research trials (research registry), (2) providing or evaluating care (service or quality registry), or (3) tracking the prevalence of dementia (epidemiological or population registry).^6^ Each type of registry supports a dementia response; however, silos for specific research trials, services, or localities create barriers to comprehensive, coordinated programming, which includes policy, public health, prevention, intervention, research, clinical and social support, and education.^1^

### 2.2 Limitations in ADRD estimates

In the United States, dementia prevalence estimates are generated from various sources, including longitudinal cohort studies, Medicare claims data, the Behavioral Risk Factors Surveillance Survey (BRFSS), and census data.^9^, ^10^, ^11^ These estimates are valuable but limited by a lack of sensitivity, insufficient caregiver data, and the underrepresentation of populations with limited health care access, non–Medicare‐eligible individuals, and preclinical cases.^12^, ^13^, ^14^

State‐level dementia registries enhance Medicare and BRFSS data to strengthen state responses to dementia. The largest state registries are in Georgia (founded in 2014),^15^ West Virginia (founded in 2006),^16^ and South Carolina (founded in 1988).^17^ Statewide registries differ from other dementia surveillance because (1) they are created explicitly to monitor dementia trends within a state; and (2) they are funded, operated, and codified by a state government or partner, creating a direct avenue for states to coordinate a dementia response. The largest statewide registries rely on an automatic enrollment strategy, using claims data to partition ADRD diagnoses into the registry from a larger database. Statewide registries are highly useful; more than 340,000 ADRD cases have been identified in South Carolina using registry data.^17^ In Georgia and West Virginia, registry data have been used to estimate increases in cognitive decline, inform state policy, and help estimate health care costs.^15^ State registries are also used to support caregivers^18^ and conduct observational retrospective studies on risk factors and in special populations.^19^, ^20^

### 2.3 Focus on secondary and tertiary populations: Automatic enrollment

Registries often comprise only patients with a diagnosis. Because the VMP uses BOLD Act funding and leverages the HBI Roadmap, a different strategy was indicated due to the emphasis on risk reduction and caregiver support.^21^, ^22^, ^23^, ^24^ Expanding the registry to include caregivers and SCI provides a novel mechanism to tabulate cases of SCI, caregiving, and individuals without health care, creating a policy perspective essential to support adequate and equitable resource allocation.

### 2.4 Barriers to enrollment in ADRD registries: Opt‐in enrollment

Epidemiological registries focus on disease surveillance and translational impact in policy, intervention, and resource allocation.^25^ From an enrollee perspective, the epidemiological registry model may feel unidirectional, with no perceived patient‐level benefit to enrollment. The lack of benefits, misunderstanding of registry purpose, and historic medical mistrust all hinder enrollment in registries that use an “opt‐in” strategy, in which participants choose to enroll.^26^, ^27^ This mistrust is particularly concerning in the context of ADRD, as addressing racial, ethnic, and social inequalities that disproportionately affect underserved populations necessitates a health equity approach.^23^, ^24^, ^25^

Previous research has shown that racial and ethnic minorities express hesitation in enrolling in ADRD registries.^28^, ^29^ This hesitation, caused by the inequitable distribution and withholding of resources and many other historical and contemporary factors, undergirds racial inequities in ADRD. For instance, in Alzheimer's drug trials for lecanemab and donanemab, recruitment and sampling biases contributed to a lack of racial diversity, with less than 2% of participants identifying as Black.^30^, ^31^ This underrepresentation in research and public health surveillance has serious consequences. Despite evidence showing Black Americans are 1.5 to 2 times more likely to develop dementia than White Americans, their diagnosis rates do not reflect this increased risk.^32^ The sources of this paradox include (but are not limited to) the underrepresentation of Black Americans in dementia research studies,^30^, ^31^, ^33^ inequitable access to health care, delays in or not seeking care due to lack of access, transitory diagnosis thresholds,^32^, ^34^ and implicit bias.^23^, ^24^

The concomitant disjunctions that arise from the underrepresentation in ADRD research of disproportionately vulnerable populations and those at a higher risk of developing dementia indicate the need for more rigorous approaches to data collection. To reduce inequities, the construction of innovative and comprehensive surveillance and interventions must be congruent with and responsive to needs and challenges, as they exist in communities that are inordinately vulnerable and have higher levels of dementia risk.

## 3 SOLUTION: THE VIRGINIA MEMORY PROJECT

### 3.1 The novelty and advantage of our approach

**FIGURE 1:**
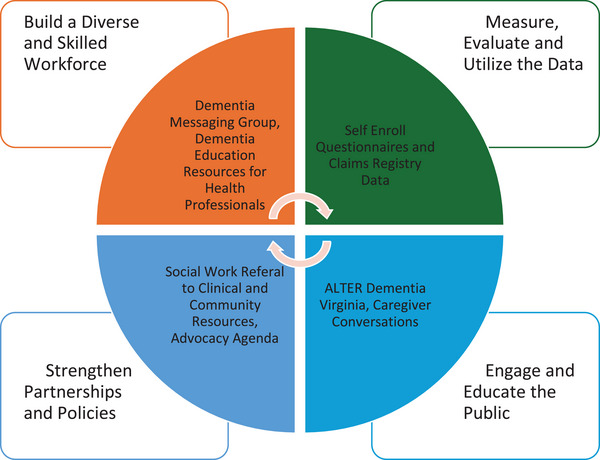
Virginia Memory Project Healthy Brain Iniative Roadmap domains.

The VMP uses the HBI Roadmap^1^ to create a new model of epidemiological ADRD registry, addressing (1) data use and evaluation, (2) workforce engagement, (3) public education, and (4) policy development. The VMP also attempts to support an equitable and coordinated response to ADRD in Virginia by mitigating enrollment barriers and providing short‐ and long‐term benefits (see Figure 1).

#### 3.1.1 Domain 1: Measure, evaluate, and utilize the data

As discussed in Sections 2.3 and 2.4, registries often use either an “automatic” or an “opt‐in” enrollment strategy. These strategies have advantages and limitations. The VMP has combined opt‐in (self‐enrollment) and an automatic claims enrollment method. Combining both conventional registry models has several advantages that support the epidemiological approach and help achieve the HBI Roadmap goals. The automatic data enrollment is partitioned from the Virginia All‐Payer Claims Database (APCD). The self‐enrollment arm is hosted on REDCap (research electronic data capture), a secure online application that creates and manages data collection methods.

##### All‐Payer Claims Database (APCD)

The APCD is a database under the authority of the VDH that collects paid medical and pharmacy claims for roughly 5 million Virginians with commercial, Medicaid, and Medicare coverage across all types of health care services. To create the VMP automatic enrollment arm, all medical claims from 2016–2022 in the APCD were queried to identify Virginia residents with any ADRD diagnosis. The onset age, year, month, and the clinical setting of the initial ADRD diagnosis were captured for each person in the registry. The VMP cataloged the ADRD year of onset, demographics, payer type, specific ADRD diagnosis identified via International Classification of Diseases, Tenth Revision (ICD‐10) codes, and flags for 17 ADRD medications identified via generic product identifier. Current Procedural Terminology (CPT) codes were also included to determine the place and type ofservice of encounter. All eligible claims of Virginia services were included in the queries.

##### Adaption of Behavioral Risk Factors Surveillance Survey (BRFSS) caregiving and cognition modules

**FIGURE 2:**
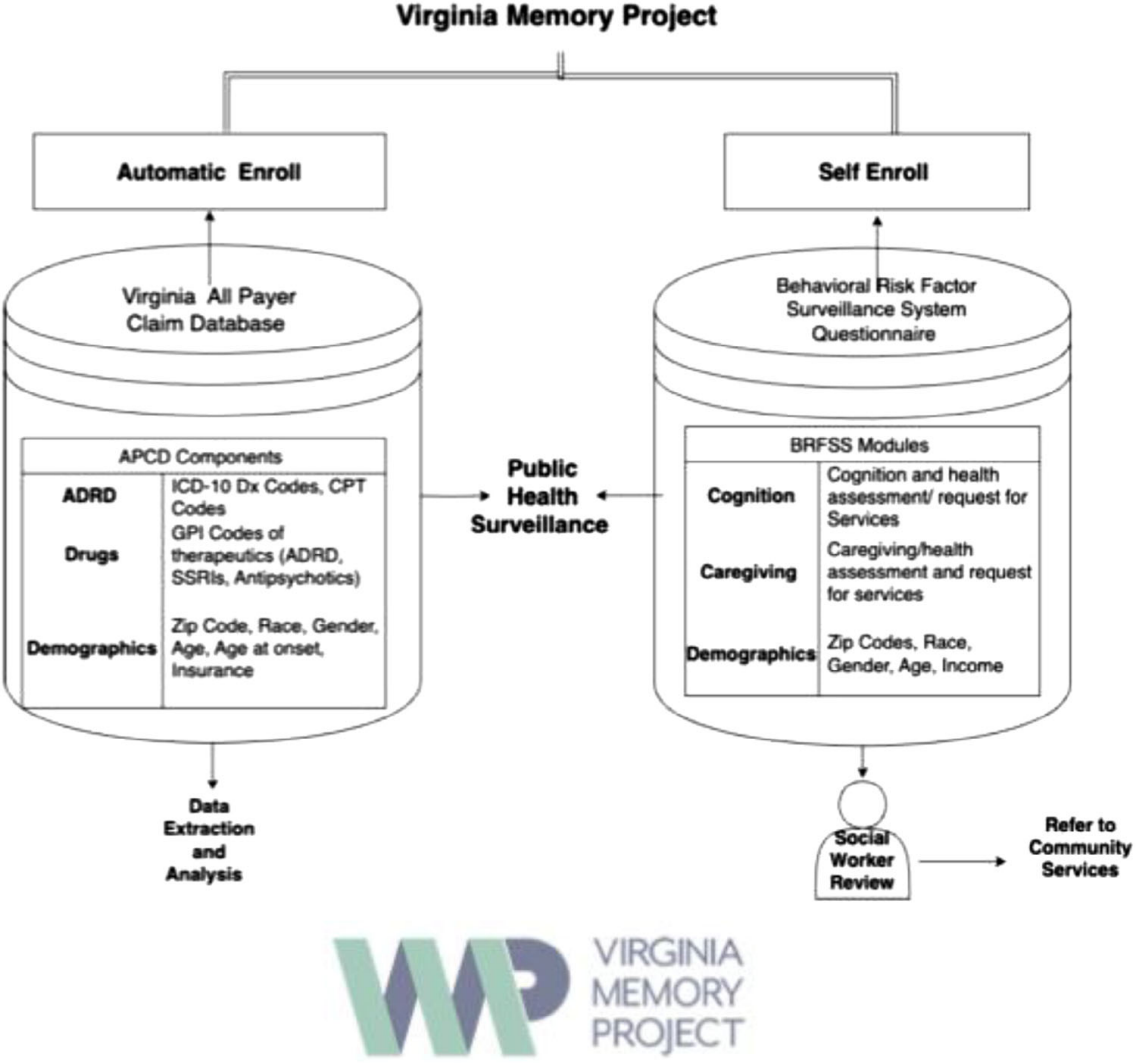
Combined data sources of the Virginia Memory Project.

The self‐enroll or “opt‐in” enrollment arm questions consist of digitized BRFSS modules on cognition, caregiving, and demographics. In addition to these questions, the self‐enroll component provides a “menu” of resources from various VMP partners, such as research trials, primary and neurology care appointments, and the local Alzheimer's Association. These responses provide a baseline for social worker review, which assists in the connection to services and provides information about the needs and concerns of individuals in Virginia. Figure 2 shows the combined data sources of the VMP.

##### *Health*Landscape Virginia

**FIGURE 3:**
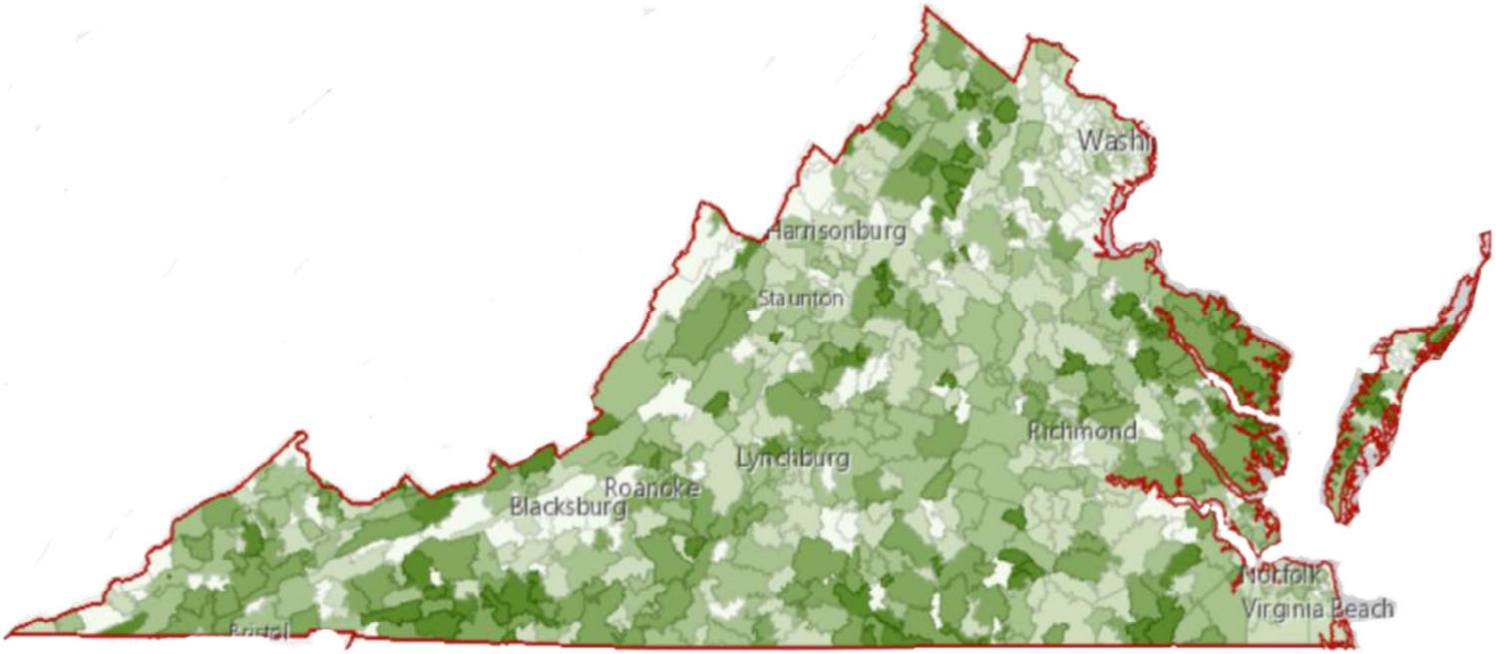
Choropleth map of Alzheimer's disease prevalence in Virginia.

Data from the Virginia APCD is adapted for public use at *Health*Landscape Virginia.^35^ In this free web‐based dashboard, users can see the prevalence of ADRD in their zip code. Additional functions include the ability to map other data, filter based on demographics, and “slice” based on other chronic diseases. By supplying the prevalence of ADRD, *Health*Landscape supports a public health response by correcting the information imbalance in which individuals lack resources to support data‐informed action in their communities. *Health*Landscape works as a tool for policy change by demonstrating the disproportionate impact of dementia in different zip codes and providing an avenue for direct and community‐engaged programming by providing organizations with information about the needs of their service area. A generated choropleth map reflecting the prevalence of Alzheimer's disease at the zip code level in Virginia generated by *Health*Landscape is shown in Figure 3.

#### 3.1.2 Domain 2: Build a diverse and skilled workforce

##### Dementia Messaging Group

In partnership with VDH, the VMP has created a “Dementia Messaging Group” (DMG) for Virginia dementia service providers. The DMG sends information, internal and external training, and resources to the dementia workforce about recent innovations, funding opportunities, continuing education, and best practices. The DMG is distinct from other dementia workforce groups because of its focus on the importance of a public health approach in dementia care and the education of dementia workforce members to support patients and clients in enrolling in the VMP. DMG providers also communicate with the VMP about their services so that VMP staff and outreach can appropriately refer enrollees to appropriate services.

##### Dementia education resources for health professionals

The VMP created an Open Education Resource (OER) to support the development of future and current health care professionals. The modules are hosted on VIVA (Virtual Library of Virginia), an online academic library, and aim to teach health care professionals and students the importance of brain health, surveillance of brain health in Virginia, and age‐friendly care practices, including non‐pharmacological interventions to address agitation and delirium. These free modules make age‐friendly dementia education easy and accessible.^36^

#### 3.1.3 Domain 3: Engage and educate the public

##### ALTER

ALTER was created in 2019 at Emory University to address the lack of resources and awareness around dementia in African American and faith communities in Georgia.^37^ Engagement in this program aims to improve the brain health status of older adults, improve physical and mental health outcomes, and reduce dementia stigma. The VMP supports the ALTER program by supplying dementia education and referral resources, including social work support. Virginia churches in the ALTER program are educated about the VMP and provided an opportunity to enroll. The partnership with the ALTER program offers a pathway to enrolling African Americans, working to increase representation, and supporting an equitable, data‐informed state dementia response.

##### Caregiver Conversations

The VMP supports “Caregiver Conversations,” a virtual, two‐session interdisciplinary “fireside chat” led by clinical practitioners. Session 1 addresses the elements of high‐quality primary care based on the American Academy of Neurology^38^ and skills for engaging with clinical team members. Session 2 focuses on caregiver support and resource utilization. Attendees are educated about the VMP at each session and given time to enroll. Responses provide an avenue for individual‐level follow‐up from the VMP social worker. In addition, responses are used to support the development of outreach and educational materials for health care professionals and community partners and in needs assessment at the local and state levels.

#### 3.1.4 Domain 4: Strengthen partnerships and policies

The VMP leverages the community voice of enrollees and connections with VDH and the State Unit on Aging to advocate for data‐informed, dementia‐capable policies in Virginia. The VMP is featured in the Virginia Dementia State Plan^39^ as critical to achieving a dementia‐capable Virginia. In 2024, HB 1455 codified the VMP with unanimous bipartisan support. In addition, the VMP has provided hundreds of Virginians with the opportunity to connect to resources, including clinical trials, clinical care, and community‐based support.

## 4 DISCUSSION

The disproportionate impact of ADRD on Black Americans and other marginalized populations is indicative of the need for and importance of increasing the granularity and accuracy of the available data on dementia and other health‐related conditions. Innovative methods, such as the VMP's enrollment strategy, are needed to support the use of the data collected to implement long‐term improvements. The VMP is a unique, statewide dementia registry applying the HBI framework to achieve substantive progress toward a coordinated dementia response in Virginia. The VMP fulfills the traditional operations of a higher‐order epidemiological registry while directly incorporating novel elements such as workforce development, community education, and empowerment into operations.

## 5 LIMITATIONS

The VMP model expands the capacity of the traditional epidemiological registry and supports HBI Roadmap objectives. However, it has limitations. Logistical and financial burdens accompany any epidemiological registry's development and subsequent maintenance.^6^ Although the VMP does not provide individualized services, the VMP's responsiveness raises costs as staff review enrollments and refer support requests.

The VMP invests significant resources in outreach, community education, and empowerment, acting as a resource hub and partnering with community agencies to support risk reduction and caregiving. Although participants in programming may benefit from learning about the VMP, enrolling, and being offered the opportunity to have a responsive follow‐up from a social worker, the self‐enroll data collected have not yet been robust or sufficiently representative to create prevalence projections. In addition, because the self‐enrollment questionnaire is based on the BRFSS questionnaires, the inherent limitations in BRFSS are also present in the self‐enrollment of the VMP, including sampling biases.^14^ The self‐enroll data provide information for educational materials, trust building, and an avenue for community members to voice needs and request ADRD supports but may have limited utility for population prevalence estimation.

Enrollment in automatic and self‐enroll databases reflects historical and contemporary inequities.^24^ In the automatic enrollment claims data partitioned from the APCD, 16.57% of ADRD diagnoses are Black or African American. In the self‐enroll component, 15.9% of enrollees identify as Black or African American.^40^ Both enrollment categories are disproportionately low compared to Virginia's population, which was 20.9% in the 2020 Census.^41^

Although the data partitioned from the APCD extend the capacity for prevalence estimation of ADRD beyond only Medicare or CDC BRFSS estimates, gaps remain. For instance, Veterans Affairs (VA) and Indian Health Service (IHS) data are not included. This missing data are significant because Veteran populations and American Indian/Alaska Native populations are populations with unique risk factors and elevated prevalence of ADRD.^42^, ^43^, ^44^ In addition to payor gaps, claims data have limitations and includes only diagnostic claims—lacking lab values, imaging results, and clinical notes. In addition, there is significant delay, with 2024 estimates currently based on 2021 data, a year heavily impacted by the coronavirus disease 2019 (COVID‐19) pandemic.^45^

## 6 NEXT STEPS

### 6.1 Increasing enrollment

The VMP's primary goal is to provide accurate and inclusive prevalence estimates to support data‐driven, community‐focused policy, resource allocation, and program evaluation in Virginia. Strategies that build trust and overcome enrollment barriers are crucial to achieving a coordinated dementia response. Future VMP work includes leveraging Community Health Workers (CHWs), who are trusted within their communities and can reach groups that traditional campaigns miss, and^46^, ^47^ partnering with family medicine practices to strengthen clinical workforce capacity to provide wrap‐around supports.^48^ Increasing self‐enrollment will provide valuable data to evaluate the feasibility of the VMP model as a responsive public health approach, particularly in enhancing access to screening and diagnostic services. Automatic enrollment will increase registry numbers via claims data, which capture emerging treatments and risk factors.^49^, ^50^, ^51^

### 6.2 Expanding and investing in data standards for caregiving

Currently, collecting prevalence data on caregivers and SCI presents multiple challenges, including self‐report bias and a lack of specificity in diagnosis, or a lack of diagnosis altogether. However, these issues may be eased in the future with updates to billing and coding, such as the Medicare GUIDE (Guiding an Improved Dementia Experience) model^52^ and increased focus on social determinants of health. For instance, adopting “Z” codes in the ICD‐10 (non‐medical factors that affect a patient's health status) will provide data on caregiving prevalence.^53^ These claims data points will generate a more robust view of the state of caregiving. The increased specificity of caregiving and dementia data standards will provide more accurate and complete data surveillance and allow more resources to be directed into outreach, trust building, and supporting individuals to get a diagnosis, thereby reducing reliance on self‐report data.

As a registry, the VMP is focused primarily on prevalence estimation but uses a variety of strategies to define and gather data into the registry. Including CHWs, caregivers, SCI, and emerging treatments and risk factors in future registry operations will support a coordinated dementia response by providing a complete modeling of the impact of ADRD in Virginia.

## 7 CONCLUSION

Policy avenues should be explored to support dementia response and the HBI Roadmap. The VMP uses a responsive philosophy and the HBI Roadmap to fulfill the surveillance requirements of a public health registry while working to overcome barriers and increase the accuracy of prevalence estimation in ADRD. These data inform educational programming, prioritize community voices, bolster direct community and clinical linkages within the VMP, and advance healthy brain legislative advocacy within Virginia. The VMP aims to mediate barriers to enrollment and surveillance by combining multiple data sources, actively engaging in educational and advocacy efforts, and providing responsive resources.

## CONFLICT OF INTEREST STATEMENT

The authors have no conflicts of interest to declare. Author disclosures are available in the supporting information.

## Supporting information



Supporting Information

## References

[1] Centers for Disease Control & Alzheimer's Association . Healthy Brain Initiative: state and local road map for public health, 2023‐2027. Centers for Disease Control; 2023.

[2] Centers for Disease Control . BOLD public health program award recipients. Centers for Disease Control; 2023. Accessed August 7, 2023.

[3] HB 1455 Virginia memory project. State of Virginia; 2024.

[4] Krysinska K , Sachdev PS , Breitner J , Kivipelto M , Kukull W , Brodaty H . Dementia registries around the globe and their applications: a systematic review. Alzheimers Dement. 2017;13(9):1031‐1047. doi:10.1016/j.jalz.2017.04.005 28576507 PMC6872163

[5] de la Paz MP , Villaverde‐Hueso A , Alonso V , et al. Rare diseases epidemiology research. Adv Exp Med Biol. 2010;686:17‐39. doi:10.1007/978-90-481-9485-8_2 20824437

[6] Pop B , Fetica B , Blaga ML , et al. The role of medical registries, potential applications and limitations. Med Pharm Rep. 2019;92(1):7‐14. doi:10.15386/cjmed-10157 30957080 PMC6448488

[7] Nichols E , Steinmetz JD , Vollset SE , et al. Estimation of the global prevalence of Dementia in 2019 and forecasted prevalence in 2050: an analysis for the Global Burden of Disease study 2019. Lancet Public Health. 2022;7(2):e105‐e125. 34998485 10.1016/S2468-2667(21)00249-8 PMC8810394

[8] Miller MC , Bayakly R , Schreurs BG , et al. Highlighting the value of Alzheimer's disease‐focused registries: lessons learned from cancer surveillance. Front Aging. 2023;4:1179275. doi:10.3389/fragi.2023.1179275 37214775 PMC10196140

[9] Bennett EE , Kwan A , Gianattasio KZ , Engelman B , Dowling NM , Power MC . Estimation of dementia prevalence at the local level in the United States. Alzheimers Dement. 2021;7(1):e12237. 10.1002/trc2.12237 PMC8719342 35005210

[10] Adams M . Estimating dementia and receipt of informal care using behavioral risk factor surveillance system data. Am J Alzheimers Dis Other Dement. 2017;32(3):129‐136. 10.1177/1533317517698792 PMC10852622 28423934

[11] Haye S , Thunell J , Joyce G , et al. Estimates of diagnosed dementia prevalence and incidence among diverse beneficiaries in traditional Medicare and Medicare Advantage. Alzheimers Dement. 2023;15(3):e12472. 10.1002/dad2.12472 PMC10450829 37636488

[12] Dhana K , Beck T , Desai P , Wilson RS , Evans DA , Rajan KB . Prevalence of Alzheimer's disease dementia in the 50 US states and 3142 counties: a population estimate using the 2020 bridged‐race postcensal from the National Center for Health Statistics. Alzheimers Dement. 2023;19(10):4388‐4395. 37458371 10.1002/alz.13081 PMC10593099

[13] Taylor DH, Jr. , Østbye T , Langa KM , Weir D , Plassman BL . The accuracy of Medicare claims as an epidemiological tool: the case of dementia revisited. J Alzheimers Dis. 2009;17(4):807‐815. 19542620 10.3233/JAD-2009-1099 PMC3697480

[14] Fahimi M , Link M , Mokdad A , Schwartz DA , Levy P . Tracking chronic disease and risk behavior prevalence as survey participation declines: statistics from the behavioral risk factor surveillance system and other national surveys. Prev Chronic Dis. 2008;5(3):A80. 18558030 PMC2483564

[15] Leonard EW , Bu R , Brown AA . Best practices for a state Alzheimer's disease registry: lessons from Georgia. University of Georgia; 2016.

[16] Abner EL , Jicha GA , Christian WJ , Schreurs BG . Rural‐urban differences in Alzheimer's disease and related disorders diagnostic prevalence in Kentucky and West Virginia. J Rural Health. 2016;32(3):314‐320. 26515331 10.1111/jrh.12155 PMC5056321

[17] Friedman D , et al. 2022 Annual report South Carolina Alzheimer's disease registry. Arnold School of Public Health; 2022.

[18] Porter CN , Miller MC , Lane M , Cornman C , Sarsour K , Kahle‐Wrobleski K . The influence of caregivers and behavioral and psychological symptoms on nursing home placement of persons with Alzheimer's disease: a matched case–control study. SAGE Open Med. Published August 23, 2016;4:205031211666187. 10.1177/2050312116661877 PMC4999794 27606063

[19] Miller M , Orwat D , Rahimi G , Mintzer J . A retrospective, population‐based cohort study of driving under the influence, Alzheimer's disease diagnosis, and survival. Int Psychogeriatr. 2019;31(4):571‐577. 30303050 10.1017/S1041610218001151

[20] Miller MC , Salgado G , Nasrallah N , Bronson J , Sabatino CP , Mintzer J . Dementia in the incarcerated population: a retrospective study using the South Carolina Alzheimer's disease registry, USA. Int J Prison Health. 2023;19(1):109‐124. 36821370 10.1108/IJPH-08-2021-0071 PMC10460458

[21] Livingston G , Huntley J , Sommerlad A , et al. Dementia prevention, intervention, and care: 2020 report of the Lancet Commission. Lancet. 2020;396(10248):413‐446. 32738937 10.1016/S0140-6736(20)30367-6 PMC7392084

[22] González‐Madrid A , Calfío C , González A , Lüttges V , Maccioni RB . Toward prevention and reduction of Alzheimer's disease. J Alzheimers Dis. 2023;96(2):439‐457. 37807781 10.3233/JAD-230454

[23] Lin P‐J , Daly AT , Olchanski N , et al. Dementia diagnosis disparities by race and ethnicity. Med Care. 2021;59(8):679. 34091580 10.1097/MLR.0000000000001577 PMC8263486

[24] Alzheimer's Association . Special report: race ethnicity and Alzheimer's in America. Alzheimer's Association. 2021.

[25] Thacker SB , Berkelman RL . Public health surveillance in the United States. Epidemiol Rev. 1988;10:164‐190. 3066626 10.1093/oxfordjournals.epirev.a036021

[26] Berry JG , Ryan P , Gold MS , Braunack‐Mayer AJ , Duszynski KM . A randomised controlled trial to compare opt‐in and opt‐out parental consent for childhood vaccine safety surveillance using data linkage. J Med Ethics. 2012;38(10):619‐625. 22518045 10.1136/medethics-2011-100145

[27] Vorstius Kruijff PE , Witjes M , Jansen NE , Slappendel R . Barriers to registration in the national donor registry in nations using the opt‐in system: a review of the literature. Transplant Proc. 2018;50(10):2997‐3009. 30577159 10.1016/j.transproceed.2018.01.054

[28] Neffa‐Creech D , Aggarwal R , Stowell C , et al. Understanding barriers and facilitators to signing up for a mobile‐responsive registry to recruit healthy volunteers and members of underrepresented communities for Alzheimer's disease prevention studies. J Prev Alzheimers Dis. 2023;10(4):865‐874. 37874109 10.14283/jpad.2023.67 PMC10884139

[29] Ashford MT , Zhu D , Bride J , et al. Understanding online registry facilitators and barriers experienced by Black brain health registry participants: the community engaged digital Alzheimer's research (CEDAR) study. J Prev Alzheimers Dis. 2023;10(3):551‐561. 37357297 10.14283/jpad.2023.25 PMC10395260

[30] Reardon S . Alzheimer's drug trials plagued by lack of racial diversity. Nature. 2023;620(7973):256‐257. doi:10.1038/d41586-023-02464-1 37532857

[31] Van Dyck CH , Swanson CJ , Aisen P , et al. Lecanemab in early Alzheimer's disease. N Engl J Med. 2023;388(1):9‐21. doi:10.1056/NEJMoa2212948 36449413

[32] Lennon JC , Aita SL , Bene VAD , et al. Black and White individuals differ in dementia prevalence, risk factors, and symptomatic presentation. Alzheimers Dement. 2022;18(8):1461‐1471. 34854531 10.1002/alz.12509 PMC9160212

[33] Allen A . Why the next big hope for Alzheimer's might not help most Black patients. KFF Health News; 2023.

[34] McGinley L . Data on new Alzheimer's drug and Black patients is sparse. Washington Post; January 29, 2024.

[35] Health Landscape Virginia . Turning health statistics into information; Updated March 2024. Accessed November 2024. https://maps.healthlandscape.org/virginia/

[36] Farkas M . Age‐friendly care. Edited by Rhodes A . VIVA Open; 2023: https://vivaopen.oercommons.org/courseware/lesson/1842

[37] Epps F , Moore M , Chester M , et al. The Alter Program: a nurse‐led, dementia‐friendly program for African American faith communities and families living with dementia. Nurs Adm Q. 2022;46(1):72‐80. 34860803 10.1097/NAQ.0000000000000506 PMC8647771

[38] Sanders AE , Nininger J , Absher J , Bennett A , Shugarman S , Roca R . Quality Improvement in neurology: Dementia management quality measurement set update. Neurology. 2017;88(20):1951‐1957. 28461640 10.1212/WNL.0000000000003917

[39] Virginia Alzheimer's Disease and Related Disorders Commission . Virginia dementia state plan 2024‐2027; building a dementia capable Virginia. Department of Aging Services; 2024.

[40] Virginia memory project (Database). Virginia: Virginia Center on Aging; 2022. Accessed September 13, 2024. Updated August 2023.

[41] America Counts Staff. *Virginia adds more than 600,000 people since 2010* . U.S. Census Bureau; 2020. Accessed 2024.

[42] Qureshi SU , Kimbrell T , Pyne JM , et al. Greater prevalence and incidence of dementia in older veterans with posttraumatic stress disorder. J Am Geriatr Soc. 2010;58(9):1627‐1633. 20863321 10.1111/j.1532-5415.2010.02977.x

[43] Ritchie K , Cramm H , Aiken A , Donnelly C , Goldie K . Post‐traumatic stress disorder and dementia in veterans: a scoping literature review. Int J Ment Health Nurs. 2019;28(5):1017‐1031. 31106950 10.1111/inm.12601

[44] Sehar U , Kopel J , Reddy PH . Alzheimer's disease and its related dementias in US Native Americans: a major public health concern. Ageing Res Rev. 2023;90:102027. 37544432 10.1016/j.arr.2023.102027 PMC10515314

[45] Couture A , Iuliano AD , Chang HH , et al. Estimating COVID‐19 hospitalizations in the United States with surveillance data using a Bayesian hierarchical model: modeling study. JMIR Public Health Surveill. 2022;8(6):e34296. 35452402 10.2196/34296 PMC9169704

[46] Ignoffo S , Gu S , Ellyin A , Benjamins MR . A review of community health worker integration in health departments. J Community Health. 2024;49(2):366‐376. 37828419 10.1007/s10900-023-01286-6 PMC10924716

[47] Virginia Department of Health . Community health worker‐ADRD training. Healthy Brain Virginia; 2024. Accessed 2024. https://www.vdh.virginia.gov/brain‐health/working‐with‐clients‐with‐dementia/

[48] Layne M , Rhodes A , Bluestein D . Help aging patients by focusing on ‘what matters most’. Virginian‐Pilot. March 30th, 2024. https://www.pilotonline.com/2024/03/30/column‐help‐aging‐patients‐by‐focusing‐on‐what‐matters‐most/

[49] Zhang W , Liang J , Li C , et al. Age at diagnosis of atrial fibrillation and incident dementia. JAMA Netw Open. 2023;6(11):e2342744. 37938842 10.1001/jamanetworkopen.2023.42744 PMC10632957

[50] American Academy of Neurology . Monoclonal antibodies for Alzheimer's resources. 2024. Published 2024. Accessed 2024. https://www.aan.com/tools‐resources/monoclonal‐antibodies‐alzheimers

[51] Gunes S , Aizawa Y , Sugashi T , Sugimoto M , Rodrigues PP . Biomarkers for Alzheimer's disease in the current state: a narrative review. Int J Mol Sci. 2022;23(9):4962. 35563350 10.3390/ijms23094962 PMC9102515

[52] Jönsson L , Wimo A , Handels R , et al. The affordability of Lecanemab, an amyloid‐targeting therapy for Alzheimer's disease: an EADC‐EC viewpoint. Lancet Reg Health Eur. 2023;29:100657. 37251789 10.1016/j.lanepe.2023.100657 PMC10220264

[53] Center for Medicare and Medicaid Services; GUIDE Payment Methodology Paper: Including Overview of Beneficiary Alignment July 2024 – June 2025. 2024. https://www.cms.gov/files/document/guide‐payment‐methodology‐paper.pdf

[54] Center for Medicare and Medicaid Services . Using Z Codes: The Social Determinants of Health (SDOH) data Journey to better Outcomes. 2023. https://www.cms.gov/files/document/zcodes‐infographic.pdf

